# Supplementary material for: Within-person structures of daily cognitive performance differ from between-person structures of cognitive abilities
Source: PeerJ. 2020 Jun 9;8:e9290. doi: 10.7717/peerj.9290 (PMC7292017; doi:10.7717/peerj.9290)

A

Within-Person Data (Raw) Dimension 1

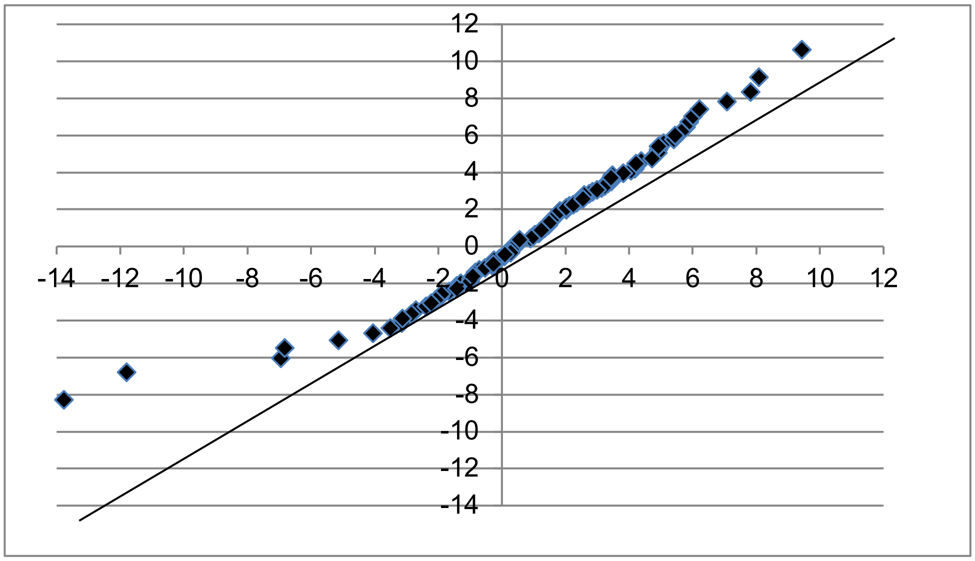

Within-Person Data (Raw) Dimension 2

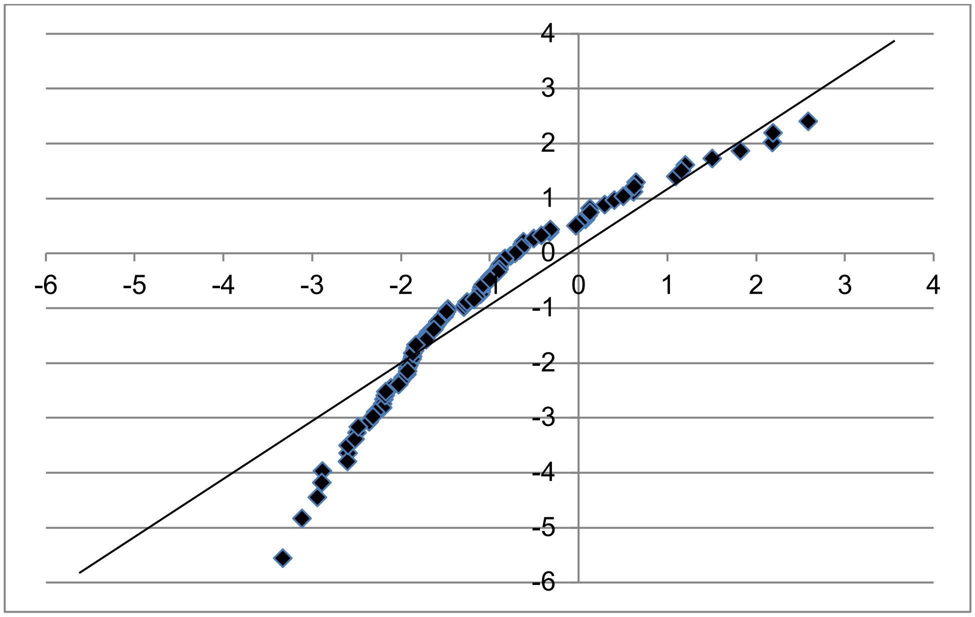

B

Within-Person Data (De-trended) Dimension 1

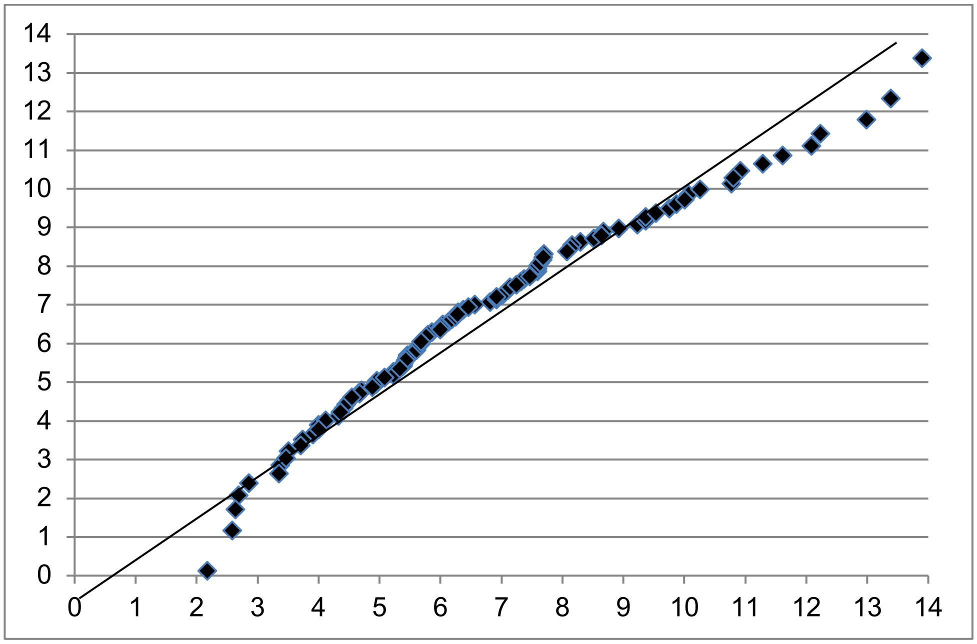

Within-Person Data (De-trended) Dimension 2

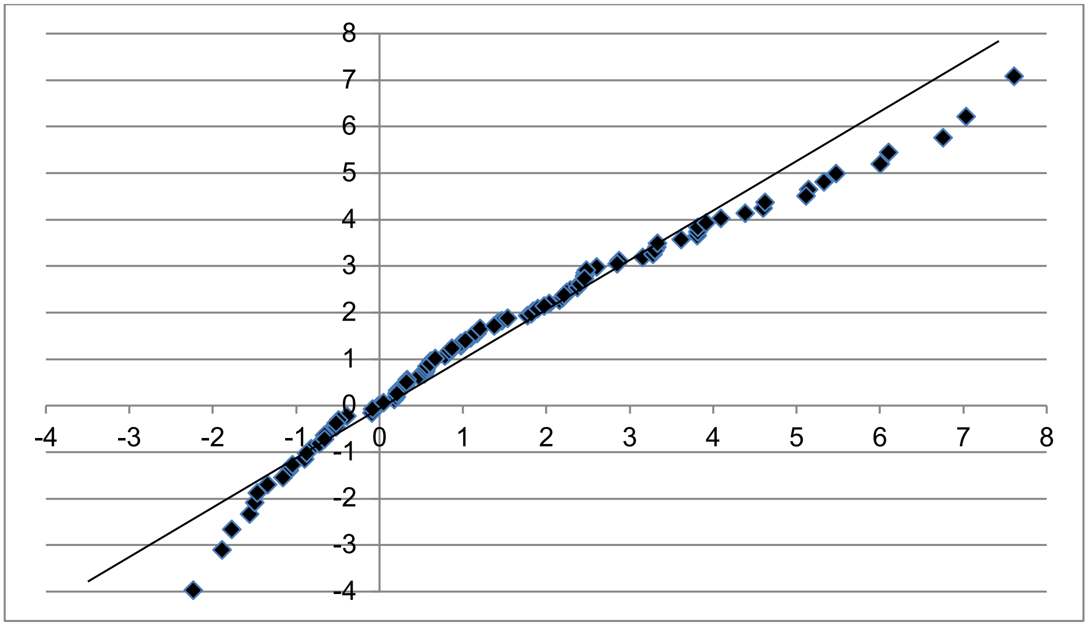

C

Between-Person Data (Pretest) Dimension 1

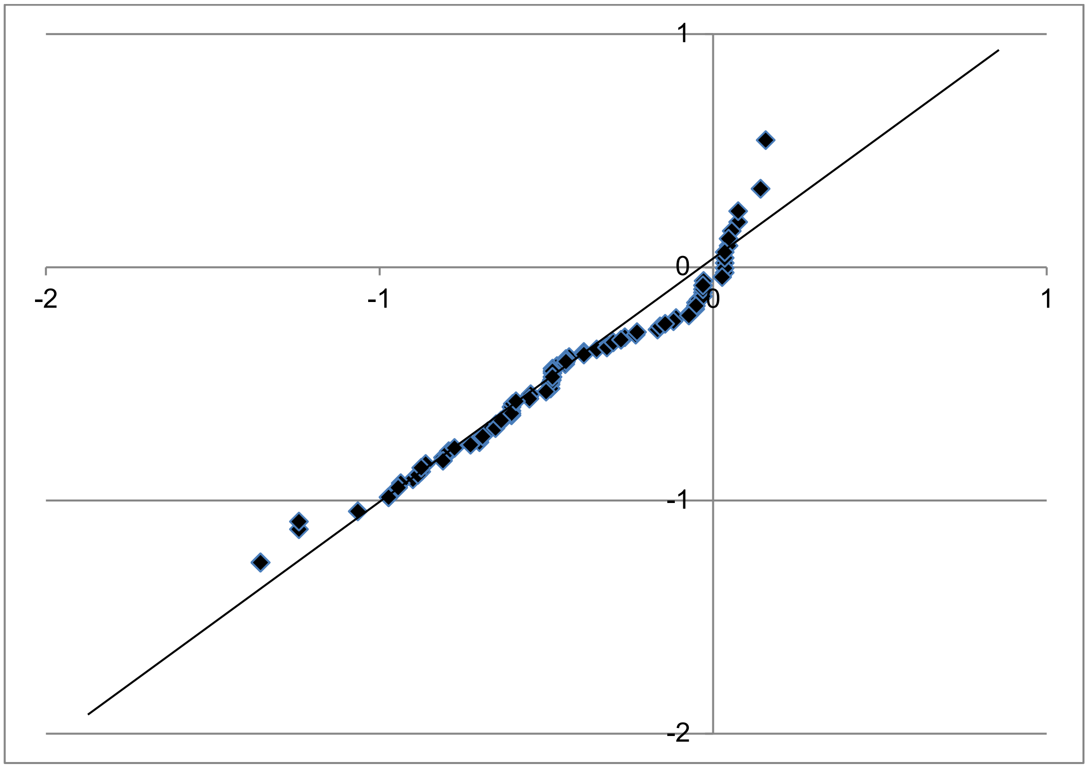

Between-Person Data (Pretest) Dimension 2

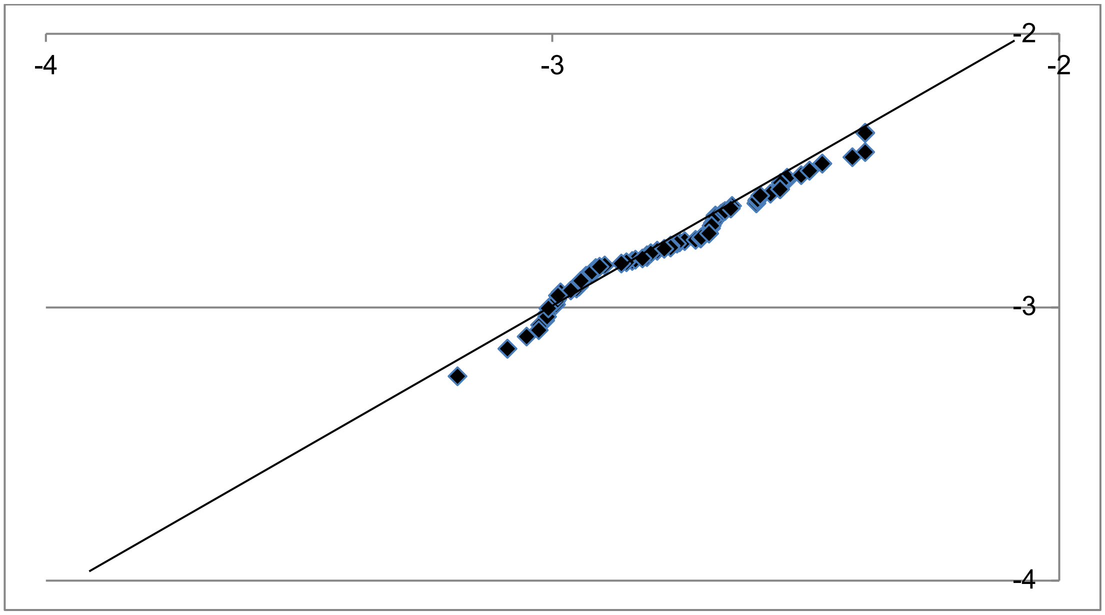

D

Between-Person Data (Posttest) Dimension 1

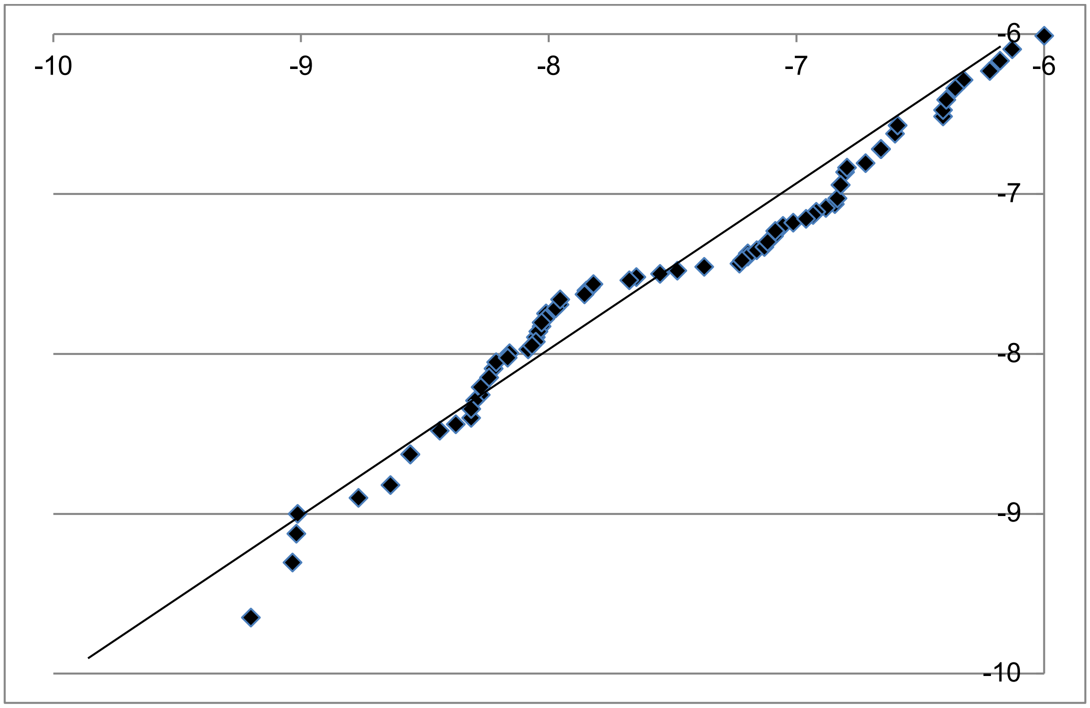

Between-Person Data (Posttest) Dimension 2

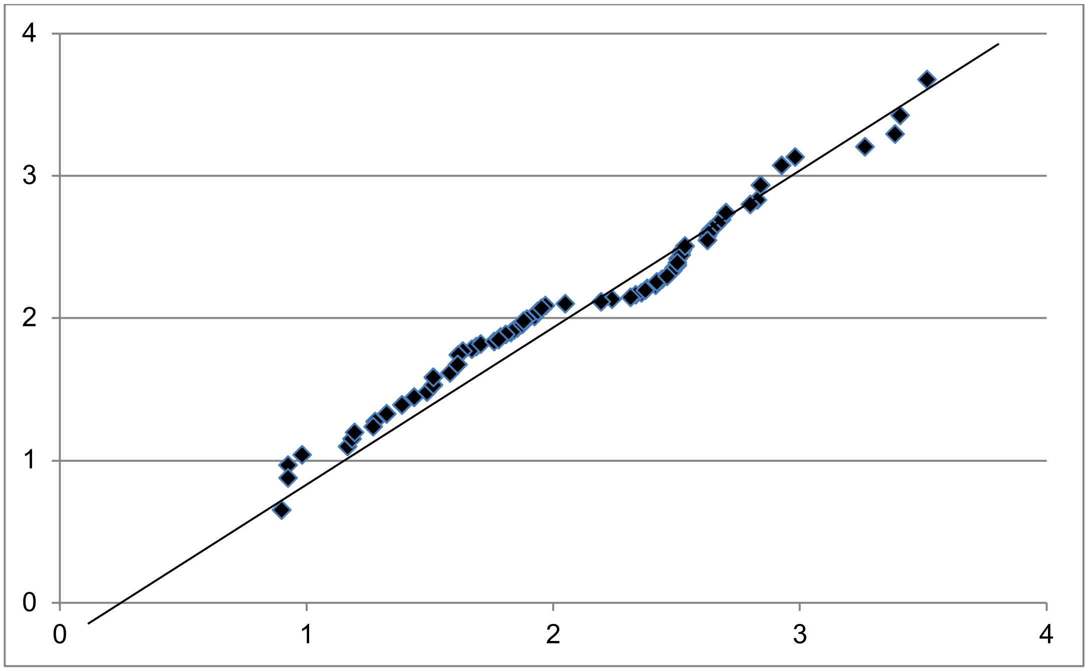

Supplement: Figure S1 [file peerj-08-9290-s001.pdf]
