# Supplementary material for: Within-person structures of daily cognitive performance differ from between-person structures of cognitive abilities
Source: PeerJ. 2020 Jun 9;8:e9290. doi: 10.7717/peerj.9290 (PMC7292017; doi:10.7717/peerj.9290)
Supplement: Table S1 — Between-person correlations below diagonal; within-person correlations above diagonal. WM1–3 = working memory tasks; PS1–3 = perceptual speed tasks; EM1–3 = episodic memory tasks. [file peerj-08-9290-s002.docx]

Table 1. *Between-Person and Within-Person Correlation Matrix of One Participant with a KL Divergence of 5.95.*

|  | PS1 | PS2 | PS3 | WM1 | WM2 | WM3 | EM1 | EM2 | EM3 |
| --- | --- | --- | --- | --- | --- | --- | --- | --- | --- |
| PS1 | 1.00 | .65 | .71 | .50 | .38 | .31 | .28 | -.11 | -.02 |
| PS2 | .76 | 1.00 | .69 | .58 | .58 | .47 | .40 | .02 | .28 |
| PS3 | .60 | .44 | 1.00 | .58 | .50 | .40 | .25 | -.18 | .13 |
| WM1 | .13 | .21 | .04 | 1.00 | .42 | .27 | .28 | .09 | .23 |
| WM2 | .10 | .13 | .15 | .08 | 1.00 | .47 | .35 | -.02 | .31 |
| WM3 | .12 | .14 | .09 | .37 | .16 | 1.00 | .36 | .25 | .02 |
| EM1 | .08 | -.02 | .02 | .26 | .18 | .22 | 1.00 | .33 | .12 |
| EM2 | .13 | .08 | -.03 | .19 | .16 | .12 | .68 | 1.00 | .10 |
| EM3 | .20 | .15 | .13 | .32 | .13 | .31 | .48 | .54 | 1.00 |

Note. Between-person correlations below diagonal; within-person correlations above diagonal. WM1–3 = working memory tasks; PS1–3 = perceptual speed tasks; EM1–3 = episodic memory tasks.
