# Supplementary material for: Within-person structures of daily cognitive performance differ from between-person structures of cognitive abilities
Source: PeerJ. 2020 Jun 9;8:e9290. doi: 10.7717/peerj.9290 (PMC7292017; doi:10.7717/peerj.9290)
Supplement: Supplemental Information 1 [file peerj-08-9290-s003.pdf]

Why to Use the KL Divergence to Compare the Distance of Multivariate Normal Distributions Described by Covariance or Correlation Matrices

The entropy of random variable X over a discrete universe Ω is the expected number of bits needed to express outcomes from this distribution. For example, a uniform distribution over two outcomes 'true' and 'false' would have an entropy of 1, because we need one bit to represent the result. If there are four outcomes the entropy would be 2 (as two bits are needed), and so on. If the distribution is not uniform, the expected numbers of bits we need to describe the result decrease, because we can assign representations (usually called *codes*) using less bits (i.e., shorter codes) to more likely outcomes and longer codes to less likely outcomes.

The entropy can be seen as describing the 'lack of information' we have about X. Scientific progress is equal to reduction of entropy in the random variables in the real world. The more we understand the world, the better we can predict future outcomes. In a normally distributed variable X, entropy is in a 1:1 relation with the variance. If a covariate explains more variance, that means the residual variance, and hence the entropy, is smaller, and we know more about the outcome.

The entropy H can be computed as

$$H(X) = -\sum_{\omega \in \Omega} P(X = x) \log(P(X = x))$$

Note that this is also the expected logarithm of the probability, $\mathbb{E}(\log(P(X))$.

If X is continuous (over real numbers), the bits we need to code outcomes of X depend on how precisely we round X (since more precise round requires more bits to represent all digits). The entropy in this case is defined as the number of bits needed to code the outcome, rounded to a small precision ϵ, minus the number of bits needed to code ϵ itself. This number actually converges to a fixed value as ϵ becomes smaller and smaller.

For example, if we round X to steps of $\frac{1}{4}$, the bits we need to code the outcomes approximately is the (continuous) entropy plus two; if we round to a precision of $\frac{1}{8}$, we need three bits more, and so on. So the entropy still reflects our intuition that more entropy means less knowledge about the variable.

Computationally, it is easy to show that the entropy of a continuous random variable X is again the expected log density $\mathbb{E}(\log(f))$ if f is the density. This can be written as

$$H(X) = -\int_{x=-\infty}^{\infty} f(x) \log(f(x)) dx$$

If we assume a wrong distribution g instead of the true distribution f, more bits are needed to code the outcomes of X. This loss, i.e., the number of additional bits we need if a wrong distribution g is assumed instead of f, is the Kullback-Leiber (KL) divergence of f and g. It can be computed as the difference of the expected log density g and the expected log density f,

$$KL(f, g) = \int_{x=-\infty}^{\infty} f(x)(\log(g(x)) - \log(f(x))$$

Intuitively, the KL describes how much information we loose because we assume a wrong model for X. If $f = g$, the KL divergence is zero. Note that the KL divergence can be negative, too, if $\log(f(X))$ is in expectation higher than $\log(g(X))$. For example, if the true distribution $f(X)$ has very low variance even though we prepared the code for a high-variance distribution g, we may in fact use less bits than anticipated before. Nevertheless, of course, we would have used even less bits if we prepared the code for the correct distribution f.

Note that the KL divergence is not symmetrical; if the true model is g but we wrongly assume f, the costs may differ from the inverted case. Therefore, one often uses the average of both directions (sometimes termed the symmetrical KL divergence) as the expected number of bits lost if, uniformly chosen, one density is wrongly used for the other:

$$KL_{sym}(f, g) = \frac{1}{2}\left(KL(f, g) + KL(g, f)\right)$$

If f and g both follow a normal distribution with equal means, then the KL divergence depends only on the covariance matrices of both distributions (or equivalently, on their correlation matrices), since the normal distribution is fully determined by these (cf. Tumminello, Lillo, & Mantegna, 2007). So the KL divergence, via the underlying distribution, also serves as a distance measure between covariance or correlation matrices. However, in this case the KL divergence still retains all properties above - it is still a comparison of distributions (the underlying normal distributions), and it still describes a lack of knowledge about the outcome, or in other terms, the scientific error we make when assuming a normal distribution with one covariance matrix if the other covariance matrix is the true underlying distribution.

Computationally, the KL divergence of two normal distributions f and g with covariance matrix Σ_1 and Σ_2 (both with K variables) and equal means is given by

$$\begin{aligned}
H(f, g) &= \int_{-\infty}^{\infty} f(x) \log\left(\frac{g(x)}{f(x)}\right) dx \\
&= \int_{-\infty}^{\infty} f(x) \log\left(\frac{|\Sigma_2|}{|\Sigma_1|}\right) - x^T(\Sigma_2 - \Sigma_1)x dx \\
&= \log\left(\frac{|\Sigma_2|}{|\Sigma_1|}\right) - \int_{-\infty}^{\infty} f(x) x^T(\Sigma_2 - \Sigma_1)x dx \\
&= \log\left(\frac{|\Sigma_2|}{|\Sigma_1|}\right) - \mathrm{Tr}\left((\Sigma_2 - \Sigma_1)\Sigma_1^{-1}\right) \\
&= \log\left(\frac{|\Sigma_2|}{|\Sigma_1|}\right) - \mathrm{Tr}\left(\Sigma_2\Sigma_1^{-1}\right) + K
\end{aligned}$$

and consequently the symmetrical KL divergence by

$$\frac{1}{2}(H(f, g) + H(g, f)) = K - \frac{1}{2}\left(\mathrm{Tr}\left(\Sigma_2\Sigma_1^{-1}\right) + \mathrm{Tr}\left(\Sigma_1\Sigma_2^{-1}\right)\right)$$

Here, we used the natural logarithm instead of the base 2 logarithm, i.e., the result is in nats rather than in bits, where 1 nat = 1.44 bits. We can easily check that the result is symmetrical, and zero if both covariance matrices are identical.

We again should keep in mind that although this is an expression in the covariance matrices, the KL still is between two normal distributions (with same mean and the two covariance matrices as parameter). Therefore, if we are interested in describing how 'wrong' we are when using one distribution where really the other should have been used, the KL is the best choice of a metric. Its result (when adding the rounding precision) can directly be interpreted in terms of bits of information that we loose when choosing the wrong covariance matrix in the normal distribution.

References

Tumminello, M., Lillo, F., & Mantegna, R. (2007). Kullback-leibler distance as a measure of the information filtered from multivariate data. *Physical Review E*(76:031123). doi: 10.1103/PhysRevE.76.031123
